# The PROMISe to increase precision in adjuvant therapy for early breast cancer: To “Type” or to “Print”?

**DOI:** 10.1038/s41523-018-0064-8

**Published:** 2018-05-22

**Authors:** Fatima Cardoso, Giuseppe Curigliano

**Affiliations:** 1 Breast Unit, Champalimaud Clinical Center/Champalimaud Foundation, Lisbon, Portugal; 2 Department of Hematology and Oncology, European Institute of Oncology, University of Milano, Via Ripamonti 435, 20141 Milano, Italy

The PROMIS trial (Prospective Study of MammaPrint in Breast Cancer Patients With an Intermediate Recurrence Score) enrolled 840 patients with early-stage breast cancer and an intermediate 21-gene assay (21-GA) recurrence score of 18 to 30. The primary aim of the trial was to assess the change in physician treatment decision before vs. after receiving the MammaPrint (also called 70-Gene Signature (70-GS)) result.^1^ Among the 840 patients who underwent 70-GS classification, 374 (44.5%) had a low-risk and 466 (55.5%) had a high-risk result. A significant change in adjuvant treatment was associated with receiving the 70-GS classification with an OR of 0.64 (95%CI, 0.50–0.82; McNemar test, *P* < .001) for all patients. Among the low-risk patients, 108 of 374 (28.9%) had chemotherapy removed from their treatment recommendation; and among the high-risk patients, 171 of 466 (36.7%) had chemotherapy added, for an overall change in treatment decision in 282 (33.6%) of patients. The authors concluded that the 70-GS provides clinically actionable information regarding patients classified as intermediate risk by the 21-GA and that clinicians may consider ordering the 70-GS for patients with an intermediate recurrence score for adjuvant therapy decisions.

Several genomic signatures exist and have been tested with a higher or lower level of evidence. The only two such tests that have undergone a prospective randomized phase 3 validation trial are MammaPrint and Oncotype, from which only the former has presented the complete primary analysis results. All these genomic tests are clinically useful mainly in luminal breast cancer subtype since, for the time being, both triple negative and HER-2-positive subtypes mandate the use of adjuvant chemotherapy. In addition, their clinical utility and cost-effectiveness are higher when used solely in cases where doubts remain regarding the benefit of adjuvant chemotherapy after evaluating all traditional clinico-pathological criteria. But how to choose between available tests? Should we “Type” or “Print” “genomic equivocal” estrogen receptor (ER) positive cases?

The MINDACT trial results clearly demonstrate the heterogeneity within ER positive HER2-negative cancers.^2^ In the high clinical/low genomic risk population who did not receive adjuvant chemotherapy (1550 patients, 48% node positive) a 94.7% 5-year DMFS (Distant-Metastasis-Free-Survival) was observed, confirming the primary hypothesis (of at least 92%) and proving that the integration of the 70-GS permits the identification of a cohort of ER-positive tumors with good prognosis with endocrine therapy alone, regardless of larger T stage and N1 status. A secondary analysis of the trial compared the outcome of high clinical/low genomic risk patients when receiving or not chemotherapy. Albeit not sufficiently powered to fully answer this randomized question, MINDACT has shown a small benefit of about 1.5% in 5-years DMFS for those who did receive chemotherapy. This difference must be put into perspective with the known short and especially long-term side effects of adjuvant chemotherapy (about 0.5% severe cardiac insufficiency and 0.5% acute leukemia). The MINDACT results allow us to put a number on the other side of the scale, helping the patient to take the final decision. Through the years, some criticisms were raised regarding the design of the MINDACT trial and consequently the interpretation of its results. The design of a trial depends on many factors: scientific, logistic, financial and ethical. An upfront and blinded randomization between traditional clinico-pathological factors and genomic test would have been both ethically and logistically unfeasible; one cannot hide from patients and their treating oncologists the pathology results. Furthermore, the clinical use of the test was always foreseen to be in conjunction with and not in place of the traditional clinico-pathological risk factors. Having as primary endpoint a direct comparison between chemotherapy vs. not exclusively in the relevant group of high clinical/low genomic risk patients would have required a far too high sample size, which would have made the trial financially and logistically impossible to run. The chosen design^3^ was a compromise and extensively discussed for over two years before the start of the trial. The very high rate of compliance in all treatment cohorts shows that both physicians and patients embraced the final design decision. Over a decade has passed since both MINDACT and TailorX were designed and recruited patients, and the overall understanding of the added value of genomic tests has improved. There is now consensus that they are best used in combination with traditional factors reflecting tumor burden i.e., tumor size and lymph node status. In fact, some genomic tests (such as Prosigna, Endopredict) are reported as a combined score of both genomic and pathology factors. A still controversial issue is the fact that the same tumor in the same patient may have a different risk assessment depending on the test used. Moreover, since risk is a continuous variable, one may argue that the genomic risk score should also be reported as a continuous variable. The definition of the high/intermediate/low cut-offs is a highly debatable issue, depending on the individual views about risk and benefit. An issue with the oncotype recurrence score that may be considered by some as problematic is the definition of three risk groups and the fact that, until we have the results of TailorX trial, it is unknown which treatment decision should be taken for the intermediate risk group,. MammaPrint provides a more straightforward result with only two risk groups (high/low), with a well-defined cut-off that has remained the same since the development of the test and is now the only genomic test with a level 1A evidence for its clinical utility regarding chemotherapy decision-making.

Adding to the points discussed above, the PROMIS trial definitely suggests to “Print” instead of “Type”. The 70-GS reclassified the 840 intermediate patients as low risk in 374 cases (44.5%) and high risk in 466 cases (55.5%). These high-risk and low-risk patients were found at every RS in the intermediate range. When analyzing the subgroup of 368 patients for whom the original physician-selected treatment recommendation (based on the intermediate 21-GA) conflicted with that indicated by the 70-GS, 279 (75.8%) of these patients had their treatment regimen changed; 108 of 142 70-GS low-risk patients who originally were recommended to receive chemotherapy had chemotherapy removed from their treatment recommendation, and 171 of 226 70-GS high-risk patients who originally were not recommended to receive CT had CT added to their treatment recommendation after the 70-GS result. Overall, 339 of 374 low-risk patients (90.6%) were recommended no adjuvant chemotherapy, and 409 of 466 high risk patients (87.8%) were recommended adjuvant chemotherapy. The change in treatment decision was the same regardless of LN status; 251 of 744 patients with LN-negative disease (33.7%) and 27 of 84 patients with LN-positive disease (32.1%) had a change to their chemotherapy treatment decision. After receiving the 70-GS result, physicians were queried in every case as to how the result influenced their confidence level regarding the chosen treatment plan: to “Type” or to “Print”? Physicians reported greater confidence in their treatment recommendations in 660 cases (78.6%) and reduced confidence in 49 cases (5.8%). The PROMIS trial has some pitfalls such as the lack of recurrence and survival data that were not collected, and inclusion criteria limited to patients with ER-positive/HER2-negative disease and the published intermediate-range RS of 18 to 30, not the TailorX intermediate RS of 11 to 25.

From all available data, we strongly support to “Print” instead of to “Type”, provided that the traditional clinico-pathological factors are also “imprinted”. From the balance between tumor biology and tumor burden, some questions remain unanswered and should be further studied such as stage 3 breast cancers with favorable pathology and low genomic signatures/scores, for whom (neo)adjuvant chemotherapy may be unnecessary.
